# Repeated Abortion Affects Subsequent Pregnancy Outcomes in BALB/c Mice

**DOI:** 10.1371/journal.pone.0048384

**Published:** 2012-10-31

**Authors:** Fang Lv, Xiangbo Xu, Shucheng Zhang, Lili Wang, Ning Wang, Bin He, Jiedong Wang

**Affiliations:** 1 Graduate School, Peking Union Medical College, Beijing, China; 2 Reproductive Physiology Laboratory, National Research Institute for Family Planning, Beijing, China; Northwestern University Feinberg School of Medicine, United States of America

## Abstract

**Aim:**

In this study, we aimed to establish a mouse model of repeated medical termination of pregnancy in order to determine subsequent outcomes.

**Methods:**

A model of mifepristone (RU 486)-induced medical abortion was established in BALB/c mice to facilitate the investigation of the impact of medical abortion on subsequent pregnancies, including litter sizes and newborn birth weights. Pregnant mice were sacrificed to examine midterm pregnancy status, investigate the frequency of fetal resorption, and measure placental function gene expression by real-time PCR and immunohistochemistry. Offspring liver mRNA was harvested for real-time PCR to determine gene expression and assess the effects of abortion on offspring development.

**Results:**

Mice subjected to 2 previous medical abortions experienced spontaneous abortions in subsequent pregnancies. Medical abortion caused reduced reproductive capacity and affected placental dysfunction, with reduced expression of tissue factor (TF) and genes encoding proteins involved in metabolic functions relevant to pregnancy, such as 11β-hydroxysteroid dehydrogenase 1/2 (11β-HSD1/2) and glucocorticoid receptor (GR). Reduced expression was also observed for platelet endothelial cell adhesion molecule-1 (CD31) and vascular endothelial growth factor (VEGF). In offspring from subsequent pregnancies, genes involved in lipid metabolism, which may enhance key lipid transcription factors, such as PPARA and PPARG, as well as GR/11β-HSD1, were downregulated in the liver. In addition, the sperm motility of the F1 males reduced.

**Conclusion:**

Repeated medical abortion impaired the reproductive function of female mice, significantly affecting the outcomes of subsequent pregnancies. The impact of repeated abortions on the offspring of subsequent pregnancies was also noteworthy and deserves further exploration. Thus, this model provides a useful means to study the mechanisms underlying the above phenomena, which will ultimately benefit the health of women and their children.

## Introduction

Medical or surgical abortion is one of the oldest, most commonly practiced, and most controversial procedures performed worldwide. The World Health Organization estimates that approximately 40–60 million induced abortions occur worldwide each year [1]. In a study of 8 European cities, women from 2 of 3 city clusters who had experienced induced abortion showed a significantly higher risk of adverse outcomes, including mid-trimester spontaneous abortion, preterm delivery, and delivering infants with low birth weight, than those who had not undergone induced abortion. In the third city cluster, induced abortion was not associated with any increased risk of adverse outcomes of pregnancy [2].

At present, medical abortion is used to terminate unwanted pregnancies at early stages. Mifepristone (RU 486) is widely used to terminate unwanted pregnancies worldwide, including in many European countries, the United States of America (USA), and China [3]–[6]. However, the safety of RU 486 is a major concern because of its reported short-term side effects [7]–[8]. Moreover, Zhu demonstrated that the gestational age at abortion, long interpregnancy intervals, and curettage with abortion may increase the risk of placental abruption [9].

Repeated abortions account for a large percentage of early pregnancy terminations, i.e., between 30% and 38% in Northern Europe [10] and in nearly 50% of cases in the USA [11]. An annual average of 8–13 million induced abortions are carried out in China, in which repeated abortions account for up to 50% [12]–[13]. The majority of those seeking repeated abortion are often young, unmarried, and plan to become pregnant again in the future [14].

Knowledge regarding the risks of abortion in subsequent desired pregnancies is scarce, and other effects of abortion on subsequent pregnancies remain an important public health concern. The increasing trend of medical abortion and repeated abortions, which have become fairly frequent in the younger population in particular, necessitates critical risk estimation. Even a small increase in complications during subsequent pregnancies may have a significant impact on public health. Development of an animal model that captures the effects of pregnancy termination on future reproductive abilities may help expand our understanding of the abortion process and holds great potential for the design and implementation of effective treatment strategies to minimize complications.

The objectives of this study are therefore to (i) establish the influence of repeated medical abortion on the outcomes of subsequent pregnancies in a mouse model and (ii) investigate the relationship between placental function and abortion during midterm gestation under the hypothesis that medical abortion affects subsequent pregnancies by impairing placental function-related gene expression. We also investigated alterations in gene expression, especially the expression of metabolism-related genes in litters from mice that had undergone 2 prior medical abortions.

## Materials and Methods

### Mice

Inbred BALB/c mice from the Institute of Medical Animal Experimental Center, Chinese Academy of Medical Sciences, were used in all experiments. Virgin female BALB/c mice (7–8 weeks old) were mated with fixed BALB/c male partners (9–12 weeks old). Successful mating was identified by the presence of the vaginal plug the next day. Experiments were performed in accordance with the Guide of the Care and Use of Laboratory Animals of the National Institutes of Health. The protocol was approved by the Ethics Committee of the National Research Institute for Family Planning, China.

### Determination of a Safe and Effective Dosage of Mifepristone for Medical Abortion

Following methods modified from the literature [15]–[16] on E8.5, BALB/c mice were treated with different single doses of mifepristone (RU 486; i.e., 0.3, 2, and 20 mg/kg, diluted with carboxymethyl cellulose [CMC]; intraperitoneal injection [IP]), with CMC as a placebo control. Vaginal smears were used to monitor vaginal bleeding. After 48 h (E10.5), the mice were sacrificed, and the uteri were dissected and sectioned to estimate the rate of abortion.

### Study Design, Medical Abortion Treatment Protocol, and Evaluation of Subsequent Pregnancy Outcomes After Abortion

**Figure 1:**
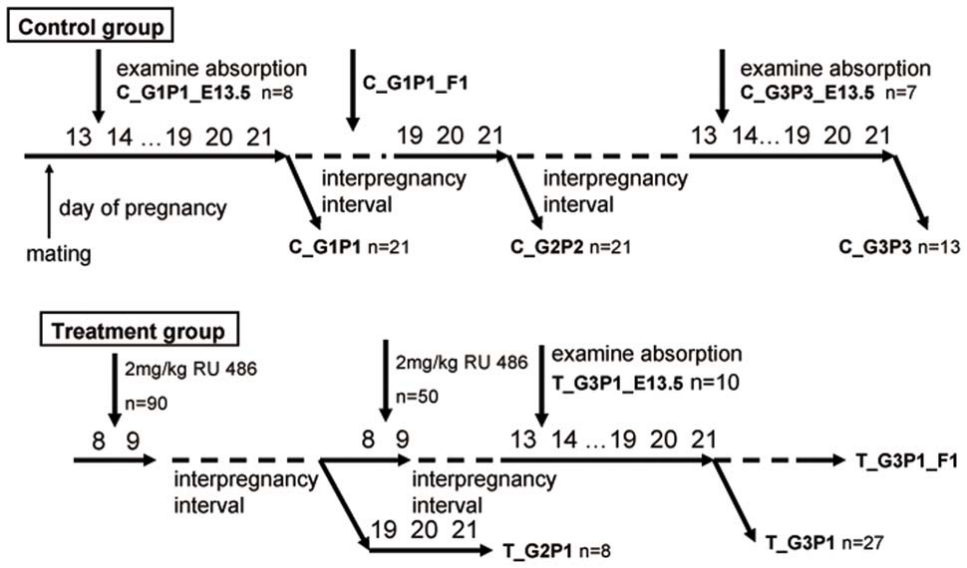
Experimental design. BALB/c mice were divided into 2 groups, the control group (C) and the abortion medicine-treated group (T). Mice in the C group were subjected to 3 normal pregnancies, termed C_G1P1 (gravida 1 and parity 1), C_G2P2 (gravida 2 and parity 2), and C_G3P3 (gravida 3 and parity 3). Mice in the T group were treated with RU 486 on day 8.5 of pregnancy. On E13.5, C_G1P1_E13.5, C_G3P3_ T_G2P1, and T_G3P1 mice were sacrificed to compare their mid-pregnancy status, the frequency of fetal resorption, and the expression of placental function-related genes. The expression of metabolism-related genes in the F1 generation (C_G1P1 mated with T_G3P1) was also compared.

Fig. 1 and Table 1 show the study design and grouping of mice, respectively. All the mice in the control and treatment groups experienced 3 pregnancy cycles. For the control group, pregnancy was allowed to proceed to term. For the treatment group, the mice were given a single dose of 2 mg/kg RU 486 on E8.5 for medical abortion and designated as group T_GxPy (where x stands for gravidity and y stands for parity) during the first 2 pregnancies. Vaginal bleeding was continuously monitored 24 h after drug administration. The third pregnancy of the treatment group (T_G3P1) was allowed to proceed to term, and the mice were carefully monitored for the entire pregnancy course. On E13.5, the treated mice (T_G3P1_E13.5) from the T_G3P1 group were sacrificed. Uteri were inspected for midgestation status, and the total numbers of implantations and resorbed sites were recorded. Fetuses, placentas, and uteri were weighed, fixed in 4% paraformaldehyde, and frozen in liquid nitrogen for further use. Similar procedures were carried out during the first and third pregnancies of the control groups, which were designated as C_G1P1_E13.5 and C_G3P3_E13.5, respectively.

**Table 1:** Experimental groups and spontaneous abortion frequencies in subsequent pregnancies after 1 or 2 abortions.

		Vaginal plug positive (0.5 dpc)	RU486 treatmentat 8.5 dpc	Successful abortions	Killed at E13.5(groups)	Term birth(groups)	Spontaneousabortion (%)	Number of offspring
Control	1st mating	50	–	–	–	21 (C_G1P1)	0	109
			–	–	8 (C_G1P1_E13.5)	–	–	–
	2nd mating	21	–	–	–	21 (C_G2P2)	0	130
	3rd mating	21	–	–	–	13 (C_G3P3)	0	84
			–	–	7 (C_G3P3_E13.5)	–	–	–
Treatment	1st mating	90	90	50	–	–	–	–
	2nd mating	50	50	37	–	–	–	–
			–	–	–	8 (T_G2P1)	62.5% (5/8)**	43
	3rd mating	37	–	–	–	27 (T_G3P1)	70.3% (19/27)** ^#^	125
			–	–	10 (T_G3P1_E13.5)	–	–	–

Notes:

A total of 140 mice were used in our experimental groups (control group, n = 50; treatment group, n = 90). We assessed spontaneous abortion by analyzing gross morphological features and vaginal smears.

** Treatment group compared with control group, *P*<0.01;

#T_G2P1 compared with T_G3P1, *P*<0.05.

For all the pregnancies that were carried to term, the litter size, birth weight, and offspring genders were recorded immediately after birth. The offspring were sacrificed by using diethyl ether and cervical dislocation at weaning (at 3 weeks of age), and the median lobe of the liver was instantaneously removed and snap-frozen in liquid nitrogen.

### Real-time PCR

Placentas from E13.5 mice (2 pups per mouse, at least 3 mice from each group) and livers from F1 offspring (8 pup livers from 4 male and 4 female mice from each group) were used for gene expression assays. Total RNA was extracted from individual frozen placenta or liver tissues with TRIzol (Invitrogen, Carlsbad, CA, USA), according to the manufacturer’s instructions. cDNA was synthesized with 2 µg of total RNA by using reverse transcriptase (RT; Takara Bio, Otsu, Japan) and was amplified using the primers listed in Table 2. Real-time PCR was performed using a StepOne Real-Time PCR System (ABI, Carlsbad, CA, USA) with SYBR Premix Ex Taq II (Takara Bio). The cycling conditions were as follows: 1 cycle for 3 min at 95°C, followed by 40 cycles (15–25 cycles for 18S rRNA) of 30-s denaturation at 95°C, 20-s annealing at 60°C, and 15-s extension at 72°C. The samples were individually amplified, and the values were averaged for each group of mice. Each PCR amplification was performed in triplicate.

**Table 2:** List of primers for real-time PCR.

Gene ID	Name	Forward primer (5′–3′)	Reverse primer (5′–3′)
ID: 17390	MMP2	GATAACCTGGATGCCGTCGTG	CTTCACGCTCTTGAGACTTTGGTTC
ID: 17392	MMP3	TTTGATGGGCCTGGAACAGTC	GGCCAAGTTCATGAGCAGCA
ID: 17393	MMP7	AGGACGACATTGCAGGCATTC	GTGAGTGCAGACCGTTTCTGTGA
ID: 17395	MMP9	GCCCTGGAACTCACACGACA	TTGGAAACTCACACGCCAGAAG
ID: 17384	MMP10	ATGAGGCTCACAACACGGACA	TTGGGTAGCCTGCTTGGACTTC
ID: 17386	MMP13	TCCCTGGAATTGGCAACAAAG	GGAATTTGTTGGCATGACTCTCAC
ID: 22339	VEGF	GTGCACTGGACCCTGGCTTTA	GGTCTCAATCGGACGGCAGTA
ID: 14254	VEGFR1	TATGTCACAGATGTGCCGAATG	CCGTAGCAGAATCCAGGTAATG
ID: 16542	VEGFR2	ACAGTCTACGCCAACCCTCC	CTCCATTCTTTACAAGCATACGG
ID: 22041	TF	CTGTTTGTTCAAGTCCACCACC	GAGCACTTCCTCATGTTACCGAC
ID: 108078	LOX-1	GTGGTTCCCTGCTGCTATGAC	CTGAGTAAGGTTCGCTTGGTATTG
ID: 15483	11β-HSD1	ATGGGAGCCCATGTGGTATTG	CAGAGGCTGCTCCGAGTTCA
ID: 15484	11β-HSD2	GTCTCCAGTGGCGACTTTCC	GTTTCTCCCAGAGGTTCACATTA
ID: 14815	GR	CTGGAATAGGTGCCAAGGG	CCATAATGGCATCCCGAAG
ID: 19013	PPARA	AAGTGCCTGTCTGTCGGGATG	CCAGAGATTTGAGGTCTGCAGTTTC
ID: 19016	PPARG	TGTCGGTTTCAGAAGTGCCTTG	TTCAGCTGGTCGATATCACTGGAG
ID: 11461	β-actin	CATCCGTAAAGACCTCTATGCCAAC	ATGGAGCCACCGATCCACA

### Immunohistochemistry

Paraffin sections (4-µm thick) were acquired according to standard protocols. A 0.3% H_2_O_2_ solution in methanol was used to block endogenous peroxidase activity for 15 min at room temperature in murine placental sections. Nonspecific staining was achieved by blocking with 10% normal serum from secondary antibody species. Sections were incubated overnight with primary antibodies CD31/VEGF (RpAb, Bioss, Beijing, China; diluted 1∶400 in PBS) at 4°C, washed in PBS 3 times for 5 min, and incubated with HRP-labeled secondary antibodies (Zhongshan, Beijing, China) for 30 min at 37°C. 3,3′-Diaminobenzidine was used as the substrate for HRP. Nuclei were counterstained with hematoxylin for 5 min. Normal goat IgG (10 mg/mL; Santa Cruz Biotechnology, Santa Cruz, CA) was used instead of primary antibodies for the negative control. The sections were dehydrated in gradient alcohol, cleared in xylene, and mounted with neutral gum.

### Reproduction Parameters for F1 Generation Male Mice

The offspring were examined until they were 15 weeks old. Adult F1 males from both the control and treatment groups were sacrificed for histological examination of the testes. Caudal epididymal sperm were counted, and their mobilities were measured.

### Statistical Analysis

The results of real-time PCR has been expressed as normalized ratios with β-actin as a reference. For mRNA quantification of each target gene, 3 independent real-time PCR experiments were performed using RNA from different extractions. Data have been expressed as the mean ± SEM values. Paired t tests were used for statistical analysis. For immunohistochemistry, at least 15 storage sections were obtained. The Mann-Whitney rank-sum test was conducted to evaluate statistical significance in immunohistochemistry experiments. Differences were considered statistically significant when *P*-values were less than 0.05 and highly significant when *P*-values were less than 0.01 (indicated by * and **, respectively).

## Results

### A Safe and Effective Dosage of RU 486 for Medical Abortion

**Figure 2:**
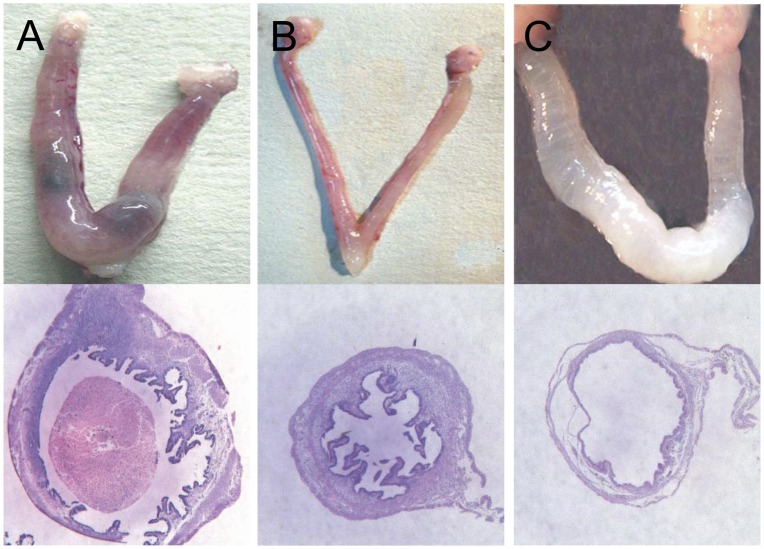
Macroscopic and microscopic structure of the uterus at 48 h after treatment with different dosages of RU 486. On day 8.5 of pregnancy, mice were treated with 3 different doses (0.3, 2, or 20 mg/kg) of RU 486. Uterine tissues were examined 48 h after the treatment. (A) Treatment with 0.3 mg/kg RU 486 caused termination of pregnancy in 60% of mice (3/5). The weight of the uterus increased, and the abortion was incomplete. (B) Treatment with 2 mg/kg RU 486 caused termination of pregnancy in 100% of cases, and the aborted horn completely regenerated. (C) Treatment with 20 mg/kg RU 486 caused pregnancy loss in 100% of mice and led to uterine dropsy (fluid retention: 3/5 mice [60%]). Therefore, the 2 mg/kg dosage was considered optimal for inducing abortion.

Three different doses of RU 486 were administered to mice at day 8.5 of the pregnancy: 0.3 mg/kg RU 486 caused pregnancy termination in 60% (3/5) of the mice, 2 mg/kg caused pregnancy termination in 100% (5/5) of mice, and 20 mg/kg caused pregnancy termination in 100% of mice and also led to uterine dropsy in 60% of the mice after 48 h (Figs. 2A, 2B, and 2C, respectively). At all 3 doses, vaginal bleeding was observed after the treatment. The 2 mg/kg dosage was considered optimal for medical abortion. Forty-eight hours after treatment with this dosage, the aborted horn completely regenerated, exhibiting normal macroscopic and microscopic structures. The abovementioned changes were not observed in the placebo group in which pregnancy was allowed to proceed to term without any signs of abortion.

### Detection of Miscarriage during Pregnancy

**Figure 3:**
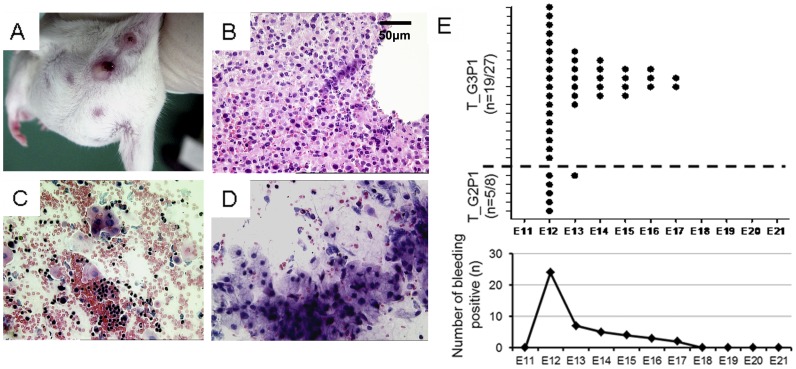
Miscarriage characteristics of subsequent pregnancies in BALB/c mice after 1 or 2 medically induced abortions. (A) Bleeding and fetal discharge from the vaginal introitus in T_G3P1 mice on day 13.5 of pregnancy. (B) The discharged blood was stained with HE. (C) Vaginal smears from the mice revealed bleeding. (D) Remnants of mesenchyme-like cells are shown. (E) Bleeding in T_G2P1 and T_G3P1 mice was observed after abortion treatment. (Scale bar, 50 µm).

Fetal discharge and bleeding were observed during midgestation in T_G2P1 and T_G3P1 mice (Fig. 3A). Large amounts of red blood cells and white blood cells and a few mesenchyme-like cells were observed by histological (Fig. 3B) and smear (Figs. 3C and 3D) examinations of the discharged blood. Bleeding due to spontaneous abortion started at E12 and lasted almost 6 days until delivery, as detected by vaginal smear analyses of T_G2P1 and T_G3P1 (T_G3P1 vs. T_G2P1 at *P*<0.05). Most instances of hemorrhage occurred at E12 in the 2 groups, and the repeated medical abortion group had a longer duration of bleeding (Fig. 3E). Spontaneous abortion frequencies in mice that had undergone 1 or 2 medical abortions were 62.5% (5/8) and 70.3% (19/27) in the T_G2P1 and T_G3P1 mice, respectively. No spontaneous abortions were observed in the control groups (Table 1).

### Size, Birth Weight, and Gender Ratio of Litters from Subsequent Pregnancies After Abortion

**Figure 4:**
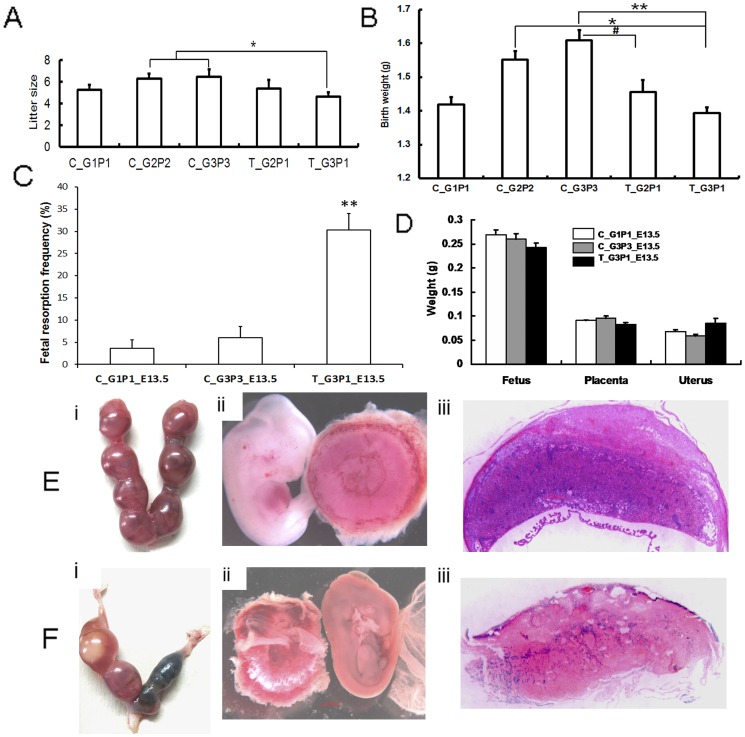
Impact of medical abortion on subsequent pregnancies. Birth weight and litter sizes of F1 mice were recorded. Litters from mice that were subjected to repeated medical abortion showed decreased size. (A) The mean size of litters from T_G3P1 mice was significantly lower than those of C_G2P2 and C_G3P3 mice (T_G3P1 vs. C_G2P2 and C_G3P3, *P*<0.05). (B) The mean birth weight of litters from T_G3P1 was less than those of C_G2P2 and C_G3P3 mice (T_G3P1 vs. C_G2P2 and C_G3P3, *P*<0.05 and *P*<0.01, respectively). The mean birth weight of T_G2P1 litters was significantly lower than that of C_G3P3 litters (*P*<0.05). (C) The pregnancy status of T_G3P1 mice was examined at 13.5 days of gestation. T_G3P1 mice had significantly increased fetal resorption frequencies compared with C_G1P1 and C_G3P3 mice (*P*<0.01). (D) The weight of the placenta, fetus, and uterus was also measured, but no statistically significant differences were observed between the control and treatment groups. (Ei and Fi) Fetal resorption was compared between the T_G3P1_E13.5 (Fi) and control group (Ei) BALB/c mice after 13.5 days of gestation. (Fii) Compared to the control groups (Eii), some placentas were abnormal, and necrosis and fetal death were observed in pregnant T_G3P1 mice. (Fiii) The placentas appeared to be small labyrinths, with degenerated structures observed in T_G3P1 mice, but not in control groups (Eiii).

After 2 medical abortions, the mean litter size from T_G3P1 mice was significantly lower than those of C_G2P2 and C_G3P3 mice (T_G3P1 vs. C_G2P2 and C_G3P3, *P*<0.05; Table 1). The mean litter size from T_G3P1 mice was also smaller than those of T_G2P1 and C_G1P1 mice, but the difference was not statistically significant (Fig. 4A). In mice that delivered their pups at term, the average birth weight of offspring in the control groups gradually increased as the number of deliveries increased over the course of 3 normal pregnancies; in contrast, the average birth weight of offspring from the T_G3P1 and T_G2P1 mice was lower than those from C_G2P2 and C_G3P3 mice (Fig. 4B: T_G3P1 vs. C_G2P2 and C_G3P3, *P*<0.05 and *P*<0.01, respectively; and T_G2P1 vs. C_G2P2 and C_G3P3, *P*>0.05 and *P*<0.05, respectively). We also evaluated the differences in birth weight of between T_G3P1 and T_G2P1 litters and found no statistically significant differences.

In terms of the gender ratio (female/male) of the offspring, T_G2P1 and T_G3P1 litters exhibited slightly higher ratios (1.07 and 1.19 vs. 1.06 in control groups); however, these differences were not statistically significant.

### Examination on Day 13.5 of Pregnancy

Next, we conducted a more detailed analysis of subsequent pregnancies in mice that had previously experienced medical abortions, in order to determine the possible reasons for miscarriages in the abortion groups. On E13.5, fetal resorption was observed in the C_G1P1, C_G3P3, and T_G3P1 mice, and T_G3P1 mice exhibited the highest frequency of resorption (*P*<0.01, Fig. 4C). From this result, we concluded that spontaneous abortion occurred during subsequent pregnancies after RU 486-induced medical abortion because of bleeding during midgestation, leading to reduction of litter sizes in mice.

Moreover, the weight of the placenta, fetus, and uterus of the nonabsorbed nodules showed no statistical difference between the control and treatment groups on E13.5 (Fig. 4D). At the resorbed sites in T_G3P1 mice, several placentas featured a small labyrinth pattern with fewer villi and contained dead fetuses (Fig. 4 Ei–iii and Fi–iii).

### Functional Gene Expression in T_G3P1_E13.5 Placentas

**Figure 5:**
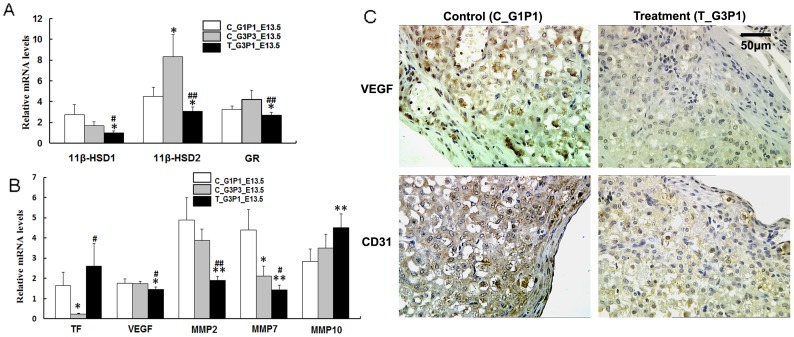
Variation in the expression of placental function-related genes. (A) Expression of metabolic genes (*11β-HSD1/2* and *GR*) and (B) genes related to placental function was detected by real-time PCR. Significant differences were observed in the expression levels of the *11β-HSD1/2*, *GR*, *TF*, *VEGF*, *MMP2*, *MMP7*, and *MMP10* genes for the control groups and the medical abortion group. (C) CD31 and VEGF expression in the placentas of the control (C_G1P1_E13.5 and C_G3P3_E13.5) and T_G3P1_E13.5 mice. T_G3P1_E13.5 tissues were stained more lightly than tissues from control groups. No staining was observed in the negative control using goat IgG. (Scale bar, 50 µm). *, *P*<0.05 vs. C_G1P1; **, *P*<0.01 vs. C_G1P1; #, *P*<0.05 vs. C_G3P3; ##, *P*<0.01 vs. C_G3P3.

The placental expression of the metabolic genes 11β-HSD1/2 and GR and the genes related to placental function was examined by qPCR analysis at E13.5 after repeated medical abortions. Significantly lower expression of 11β-HSD1/2, GR, VEGF, MMP2, and MMP7 and higher expression of TF and MMP10 were observed in the repeated medical abortion group than in the control groups on E13.5 (Figs. 5A and 5B). No differences were observed in the expression of VEGFR1, VEGFR2, PPARG, MMP3, MMP9, MMP13, and LOX-1 (data not shown). We postulated that after repeated medical abortions, changes in the expression of genes involved in certain metabolic functions in the placenta may affect offspring development.

CD31 and VEGF expression were assayed by immunohistochemistry. Weak staining of VEGF (*P*<0.05) and CD31 (*P*<0.05; Fig. 5C) was observed in placentas of G3P1_E13.5 mice. T_G3P1 mice showed reduced effective placental vascular epithelial marker expression, while the expression of VEGFR1 and VEGFR2 did not differ between control and treated groups (data not shown). The negative control (goat IgG only) showed no staining.

### Repeated Medical Abortion Affected the Expression of Metabolic Genes in the Livers of Offspring

**Figure 6:**
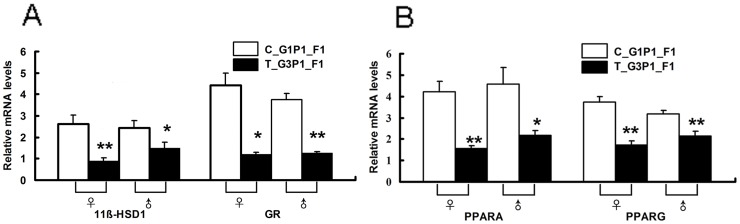
Expression of metabolism-related genes in the livers of F1 mice. The metabolism-related genes *PPARA*/*PPARG* (A) and *11β-HSD1*/*GR* (B) exhibited significantly different expression levels in the livers of C_G1P1 and T_G3P1 F1 mice. C_G1P1, n = 8 (♀:♂, 4∶4), T_G3P1, n = 8 (♀:♂, 4∶4). Assays were performed individually for each animal sample. *, *P*<0.05 vs. C_G1P1_F1; **, *P*<0.01 vs. C_G1P1_F1.

We further focused on genes related to lipid metabolism in the offspring. Downregulation of the genes involved in metabolic functions were observed in the offspring of mice that had undergone 2 medical abortions. 11β-HSD1, GR (Fig. 6B), and PPARG were downregulated in T_G3P1_F1 mice. Most notably, expression of a putative activator of the major lipid regulator PPARA also decreased (Fig. 6A). PPARD and VEGF expression showed no statistical difference between the 2 parities (data not shown).

### Abortion Decreased Sperm Motility in F1 Generation Males

**Figure 7:**
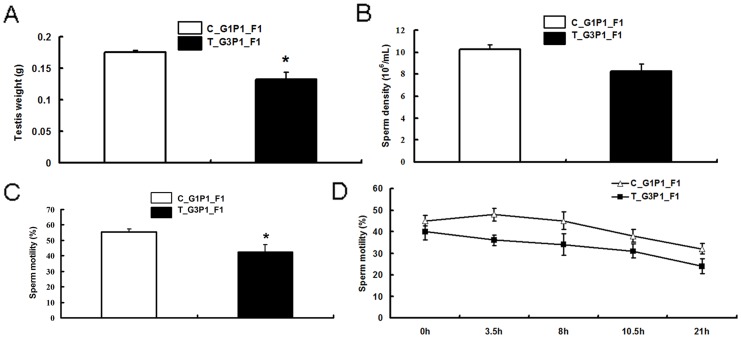
Repeated medical abortion decreased sperm motility in F1 mice. (A) T_G3P1_F1 mice had significantly lighter testicles (*P*<0.01) than C_G1P1_F1 mice at the same age. (B) No significant difference was in sperm density was observed. (C) T_G3P1_F1 mice showed decreased sperm motility (*P*<0.05) compared to control mice. (D) In vitro sperm motility plotted as a function of time showed that sperm viability in T_G3P1_F1 mice (n = 15) was decreased compared to control C_G1P1_F1 mice (n = 15; *P*<0.01).

T_G3P1_F1 mice had lighter testicles (*P*<0.01) compared with C_G1P1_F1 mice at the same age (Fig. 7A). No significant differences were found between the sperm density in the 2 groups (Fig. 7B). F1 generation offspring from mice that had undergone repeated medical abortions had decreased sperm motility (Fig. 7C). Moreover, the in vitro viability of sperm from T_G3P1_F1 mice, plotted as a function of time, decreased compared with that of the control (*P*<0.01; Fig. 7D). No difference was observed between the 2 F1 groups with respect to the gross weight of F1 mice.

## Discussion

BALB/c mice that experienced repeated early medical abortions exhibited spontaneous abortion and pregnancy loss during subsequent pregnancies. In addition, the litter sizes and birth weights of newborns from subsequent pregnancies were also adversely affected. Moreover, several changes were observed in the expression of genes involved in metabolic functions relevant to midgestation-stage placental function.

The higher frequency of spontaneous abortions in mice that have undergone repeated medical abortions than in those that had experienced only a single medical abortion suggests that the risk of spontaneous abortion increases with the increasing number of medical abortions. We also evaluated birth weights and litter sizes, but found no statistically significant differences between the 2 groups. However, mice that had experienced 2 abortions tended to have worse term birth outcomes than those that had experienced only 1 abortion. This result needs to be validated using a large sample size in future studies.

During midgestation, repeated medical abortion significantly increased fetal resorption. Similarly, studies in humans showed that abortion is associated with significantly increased occurrence of low birth weight and preterm birth, a risk that increases with the number of induced pregnancy terminations [17]–[18]. Moreover, in subsequent pregnancies, induced abortion is associated with an increased risk of first-trimester miscarriage in subsequent pregnancies [19]–[20]. Specifically, mifepristone-induced abortion increases the risk of vaginal bleeding during the early gestation period, as compared with women who have not experienced abortion [21]. All of these clinical phenomena were mimicked in the present mouse model.

In our model, negative outcomes during subsequent pregnancies occurred after 2 medical abortions, and the fetal resorption frequency significantly increased during midgestation. Fetal survival and growth depend on adequate placental function [22]–[24]. Placenta forms the interface between maternal and fetal circulation, facilitating metabolic and gas exchanges as well as fetal waste disposal. Thus, we postulated that previous medical abortions may affect placental function, and our studies focused on further investigating the expression of placenta function-related genes during midgestation.

Upon placenta and fetal injury, the TF gene is upregulated in women with recurrent miscarriages and intrauterine growth restriction [25]–[27]. In our mouse model, TF was highly expressed in T_G3P1_E13.5 mice, indicating insufficient placental function after experiencing repeated abortion. We focused on changes in previously reported panels of genes that encode proteins involved in metabolic functions relevant to pregnancy. The gene expression alterations found in our data, for example, GR and 11β-HSD2, were consistent with previous reports [28]–[29]. Glucocorticoid metabolism is important in placental function, and 11β-HSD plays an important regulatory role in this process. The two 11β-HSD isoforms, 11β-HSD1 and 11β-HSD2, have unique properties and powerful biological roles [30]–[31]. 11β-HSD1 is expressed in the decidua, placenta, lungs, spleen, kidney medulla, cerebellum, pituitary, and liver [32]. High expression of 11β-HSD2 is observed in the placenta, and this protein has been shown to play an important role in shielding the developing fetus [33]–[34]. Chronic stress reduces the activity of 11β-HSD2 [35]. Moreover, 11β-HSD2 expression is related to GR expression, which mediates 11β-HSD2 activity with proper glucocorticoid exposure in utero [36]. In our repeated abortion model, expression of both 11β-HSD1/2 and GR decreased significantly, indicating the dysfunction of placental nutrient exchange. Additionally, in previous studies, it has been hypothesized that reduced placental 11β-HSD2 activity results in high levels of glucocorticoids reaching the fetus, which retards growth and programmed disease susceptibility. Therefore, the protein level and bioactivity of 11β-HSD2, as well as the glucocorticoid levels on both sides of the placental barrier, need to be examined in further studies using the currently described model. In addition, CD31 and VEGF, primary angiogenic molecules, stained weakly in the midgestation placenta of mice subjected to repeated medical abortions. This indicated reduced placental vascular function and angiogenesis. Consistent with the above results, the placental dysfunction in mice experiencing spontaneous abortions suggested possible impairment of the maternal uterine environment. Although these were preliminary results, the changes observed in the expression of these genes warrant more detailed studies in the future.

Apart from placental dysfunction, we also explored the possibility of abnormal development of F1 generation offspring from mice subjected to repeated medical abortions. The expression of the genes involved in fat and glucocorticoid metabolic processes in the liver was analyzed. PPARs play major roles in various aspects of energy metabolism, inflammation, and offspring development [37]–[41]. Moreover, GR^−/−^ mice die within a few hours of birth because of respiratory failure, and newborn livers have reduced capacity to activate genes encoding key gluconeogenic enzymes [42]. GR is expressed in most fetal tissues, including the placenta, from early embryonic stages. Therefore, GR is essential for survival [42] and offspring development. Downregulation of PPARA, PPARG, GR, and 11β-HSD1 in the livers of offspring from the repeated-medical-abortion group suggested that this induced procedure affected offspring development, although we could not rule out the possibility that the changes in the expression of hepatic genes were due to global physiological changes caused by downregulation in some other tissue. In the present study, only comparative data between T_G3P1 and C_G1P1 were presented. Since differences in birth parameters were more significant between T_G3P1 and C_G3P3 mice (Fig. 4A, B, and C), more severe effects on F1 offspring are anticipated and should be examined further.

Mice that had undergone 2 medical abortions often experienced spontaneous abortions of subsequent pregnancies between E12 and E18, detected by virginal smears; this gestational period is a critical time in gonadal sex determination (corresponding to E8–E15 in rats) [43]–[46]. Such abortions could induce an adult phenotype exhibiting decreased spermatogenic capacity and male infertility. In the current mouse model, we studied the reproductive health of offspring and found no clear differences in the F1 male testicular structures of individuals in the repeated medical abortion group. However, 1 male mouse in the group had small testicles and a small perineal complex. Infertility was noted at 15 weeks, and the testicle was azoospermic, with a significantly altered cellular structure. Spermatogenic epithelium became thinner in some seminiferous tubules. No gross abnormalities were observed in any of the other tissues examined.

Many studies have shown that maternal experiences have transgenerational environmental effects (termed “maternal effects”) [29], [47], including in utero passage of photoperiod information in rodents [48]; inheritance of stress responses and maternal grooming behaviors in rats [34], [49]; psychiatric sequelae of fetal undernourishment in humans and rodents [29], [50]–[51]; and reprogramming of the germ line in gestating rats after transient exposure to endocrine disruptors during the gonadal sex determination period, resulting in transgenerational disease [43]. Likewise, repeated abortions may impact the environment of the maternal uterus, and such environmental changes are likely to induce an epigenetic transgenerational phenotype. Consistent with this, we observed downregulation of genes involved in metabolic functions in the liver and reduced sperm mobility in F1 mice delivered by mice that had undergone repeated abortions. DBA/2-mated female CBA/J mice represent a well-studied model of immunologically mediated pregnancy loss [52]–[53]. These mice spontaneously develop numerous features of human reproductive diseases that correlate with negative pregnancy outcomes. In these abortion-prone matings, anaphylotoxin C5a generation and increased TF expression cause dysregulation of angiogenic factors and abnormal placental development. Diminished giant trophoblast cells and placental perfusion were observed, except in negative pregnancy outcomes [54]–[55]. In the present study, we showed that BALB/c mice that experienced repeated medical abortions during midgestation had spontaneous miscarriages that shared many similar features with CBA/J×DBA/2 mice. For instance, litter size and neonate birth weight increased with parity in 3 normal pregnancies in the 2 mouse models [56]. More importantly, our model mimicked medically induced, early repeated pregnancy loss often observed in actual clinical abortion. Hence, our model is appropriate for further exploration of human reproductive diseases induced by repeated medical abortions.

Taken together, our findings established an inbred model system for investigating the impact of repeated first-trimester mifepristone-induced abortions, which led to subsequent pregnancy loss, affected the expression of placental function-related genes, and impaired development of the F1 generation. These findings suggest that an adverse fetal environment develops after repeated abortions. Additionally, repeated medical abortions may affect endometrial function, which could then impair proper placentation during subsequent pregnancies, when epigenetic regulation may be important; these hypotheses require further investigation. The present mouse model will allow us to elucidate the mechanisms involved in repeated medical abortions and will provide health benefits for women and their children.
